# Reconstruction Method for Optical Tomography Based on the Linearized Bregman Iteration with Sparse Regularization

**DOI:** 10.1155/2015/304191

**Published:** 2015-09-01

**Authors:** Chengcai Leng, Dongdong Yu, Shuang Zhang, Yu An, Yifang Hu

**Affiliations:** ^1^Key Laboratory of Nondestructive Testing of Ministry of Education, School of Mathematics and Information Sciences, Nanchang Hangkong University, Nanchang 330063, China; ^2^State Key Laboratory of Management and Control for Complex Systems, Institute of Automation, Chinese Academy of Sciences, Beijing 100190, China; ^3^Sino-Dutch Biomedical and Information Engineering School, Northeastern University, Shenyang 110819, China; ^4^School of Computer and Information Technology, Beijing Jiaotong University, Beijing 100044, China

## Abstract

Optical molecular imaging is a promising technique and has been widely used in physiology, and pathology at cellular and molecular levels, which includes different modalities such as bioluminescence tomography, fluorescence molecular tomography and Cerenkov luminescence tomography. The inverse problem is ill-posed for the above modalities, which cause a nonunique solution. In this paper, we propose an effective reconstruction method based on the linearized Bregman iterative algorithm with sparse regularization (LBSR) for reconstruction. Considering the sparsity characteristics of the reconstructed sources, the sparsity can be regarded as a kind of *a priori* information and sparse regularization is incorporated, which can accurately locate the position of the source. The linearized Bregman iteration method is exploited to minimize the sparse regularization problem so as to further achieve fast and accurate reconstruction results. Experimental results in a numerical simulation and *in vivo* mouse demonstrate the effectiveness and potential of the proposed method.

## 1. Introduction

Optical molecular imaging provides the promising tools to monitor physiological and pathological activities at cellular and molecular levels and has become an important technique for biomedical research. Meanwhile, it also has attracted attention due to its high sensitivity and low cost and has been applied to disease diagnosis, tumor detection, and drug development [1–4]. To overcome the limitation of planar imaging, bioluminescence tomography (BLT) [5], fluorescence molecular tomography (FMT) [6], and Cerenkov luminescence tomography (CLT) [7] were developed to determine the 3D distribution inside a phantom or a small animal with the reconstruction algorithm from the signal detected on the external surface associated with anatomical structure and optical properties [8].

In mathematics, one of the main challenging problems for the above modalities is severely ill-posed inverse problem, which is mainly caused by insufficient measurement and high diffusive nature of the photon propagation in tissues [9, 10]. In order to obtain a unique solution, many reconstruction algorithms have been developed and applied to linear inverse problem such as multispectral measurement [11, 12] and permissible source region (PSR) [13, 14]. These methods improve reconstruction results to a certain degree and in turn impose a variety of limitations on practical applications. The multispectral methods have some limitations by increasing measurable information [15], and both the size and position of the PSR have significant impact on the reconstruction results [16].

In addition, some regularization methods have been introduced to enhance the numerical stability and efficiency for reconstruction. The most popular is Tikhonov regularization (*l*_2_-norm) used to solve the linear inverse problem [17], which can produce oversmooth solutions. Recently, sparse regularization (*l*_1_-norm) and total variation (TV) regularization are commonly used in reconstruction. Sparse regularization has received more attention for reconstruction, which allows high quality images to be reconstructed from a small amount of measurements [18–20]. Total variation was first introduced by Rudin et al. [21] for image denoising. Now, TV regularization methods have been widely used for reconstruction [22, 23]. Meanwhile, TV regularization is effective for reconstruction because the source distribution can be taken as a nearly piecewise constant when photons are collected [24]. In order to solve these regularization methods, some novel solution algorithms were proposed such as the conjugate gradient method [25] and Split Bregman method [26, 27]. But the accuracy and efficiency of reconstruction are still challenging for the above methods. In addition, TV regularization can also produce the staircase effect.

In order to further resolve these problems, we propose a new method based on the linearized Bregman algorithm to solve the sparse regularization problem for reconstruction. The sparse regularization method can balance the merits of the sparsity characteristics and accurately locate the position of the source. In view of reconstruction accuracy and efficiency, the sparse regularization-based reconstruction problem is solved effectively by the linearized Bregman iterative algorithm. The main purpose of this paper is to show that the proposed algorithm is a very simple but very fast and accurate method in both theory and practice for reconstruction problem involving only matrix multiplication and scalar shrinkage. The experimental results including numerical simulation and an* in vivo* mouse were employed to evaluate the performance of the proposed method.

This paper is organized as follows.  Section 2 presents the linear equation and sparse regularization. Then, we give the linearized Bregman iterative algorithm to solve the sparse regularization problem.  Section 3 gives the numerical simulation and* in vivo* results to verify the performance of the proposed method and we draw the conclusion and describe further research in Section 4.

## 2. Methods

### 2.1. Linear Equation

The radiative transfer equation (RTE) is used to describe photon propagation in biological tissues belonging to the forward problem, and how to develop reconstruction algorithms to detect the internal targets or source distribution is exactly the same for the above modalities to solve the inverse problem [10]. In addition, the detailed forward problem can be found for different modalities in [28, 29].

Given the optical properties of the tissues, the solving domain can be discretized based on the finite element method (FEM) and a series of transformations and rearrangements are made for the elements in the matrix [30]. Therefore, the reconstruction problem can be simplified by the following linear relationship in the heterogeneous medium as follows:

(1) $$\begin{array}{c} AS=\Phi , \end{array}$$

where *A* ∈ *R* ^*m*×*n*^ is an ill-conditioned system matrix, Φ ∈ *R* ^*m*^ is the measured boundary flux, and *S* ∈ *R* ^*n*^ is the unknown source density.

### 2.2. Sparse Regularization

Due to insufficient measurement and the highly diffusive nature of photon propagation in tissues, (1) is an ill-posed inverse problem. In order to obtain a unique solution, we exploited the sparse regularization method to determine the source power density *S* by transferring (1) to minimize the following objective function as follows:

(2) $$\begin{array}{c} J_{\mu }\left(S\right)=\underset{S}{\min\;}\mu {\left\|S\right\|}_{1}+\frac{1}{2}{\left\|AS-\Phi \right\|}_{2}^{2}, \end{array}$$

where *μ* is the positive regularization parameter balancing the data fidelity and the regularization term ‖*S*‖_1_. The object function of (2) is convex and nondifferentiable and we will give the linearized Bregman method to solve this kind of convex optimization problem.

### 2.3. Linearized Bregman Method with Sparse Regularization

The Bregman iterative algorithm is based on Bregman Distance [31, 32], so the Bregman Distance of a convex function *E* between points *u* and *v* is defined as

(3) $$\begin{array}{c} D_{E}^{p}\left(u,v\right)=E\left(u\right)-E\left(v\right)-\langle p,u-v\rangle , \end{array}$$

where *p* is in the subgradient of *E* at the point *v*. Again, consider convex energy function *E* and convex and differentiable energy function *H* defined over *R* ^*n*^, and thus the associated unconstrained general minimization problem is given as

(4) $$\begin{array}{c} \underset{S}{\min}\left(E\left(S\right)+H\left(S\right)\right). \end{array}$$

The above problem is solved by the Bregman iterative algorithm:

(5) $$\begin{array}{c} S^{k+1}=\underset{S}{\min\;}D_{E}^{p}\left(S,S^{k}\right)+H\left(S\right). \end{array}$$

Then, (5) is approximated by adding a penalty term (1/2*δ*)‖*S* − *S* ^*k*^‖^2^ to obtain the following iterative equation:

(6) $$\begin{array}{c} S^{k+1}=\arg\underset{S}{\min\;}D_{E}^{p^{k}}\left(S,S^{k}\right)+H\left(S^{k}\right)+\langle \nabla H\left(S^{k}\right),S-S^{k}\rangle +\frac{1}{2\delta }{\left\|S-S^{k}\right\|}_{2}^{2}, \end{array}$$

where parameter *δ* is positive and serves as the step size.

The iteration equation (6) is equivalent to the following iteration by omitting the constant term with respect to *S*:

(7) $$\begin{array}{c} S^{k+1}=\arg\underset{S}{\min\;}D_{E}^{p^{k}}\left(S,S^{k}\right)+\frac{1}{2\delta }{\left\|S-\left(S^{k}-\delta \nabla H\left(S^{k}\right)\right)\right\|}_{2}^{2}. \end{array}$$

Let *H*(*S*) = (1/2)‖*AS* − Φ‖_2_^2^ by (7), and thus we have

(8) $$\begin{array}{c} S^{k+1}=\arg\underset{S}{\min\;}D_{E}^{p^{k}}\left(S,S^{k}\right)+\frac{1}{2\delta }{\left\|S-\left(S^{k}-\delta A^{T}\left(AS^{k}-\Phi \right)\right)\right\|}_{2}^{2}. \end{array}$$

Since *p* ^*k*+1^ ∈ ∂*E*(*S* ^*k*+1^) at this location, we have

(9) $$\begin{array}{c} p^{k+1}=p^{k}-\nabla H\left(S^{k+1}\right)=p^{k}-\frac{1}{\delta }\left(S^{k+1}-\left(S^{k}-\delta A^{T}\left(AS^{k}-\Phi \right)\right)\right). \end{array}$$

This yields the following iteration form from (9) due to *p* ^0^ = 0 and *S* ^0^ = 0:

(10) $$\begin{array}{c} p^{k+1}=p^{k}-A^{T}\left(AS^{k}-\Phi \right)-\frac{\left(S^{k+1}-S^{k}\right)}{\delta }=\cdots =\overset{k}{\underset{j=0}{\sum }}A^{T}\left(\Phi -AS^{j}\right)-\frac{S^{k+1}}{\delta }. \end{array}$$

Now, we consider the case of (8) when *E*(*S*) = *μ*‖*S*‖_1_ and *μ* is the regularization parameter. Then, let

(11) $$\begin{array}{c} v^{k}=\overset{k}{\underset{j=0}{\sum }}A^{T}\left(\Phi -AS^{j}\right). \end{array}$$

The linearized Bregman algorithm is given after rearrangement to solve (8), which is equivalent to solving the objective function equation (2) as follows:

(12) $$\begin{array}{c} v^{k+1}=v^{k}+A^{T}\left(\Phi -AS^{k}\right),\\ S_{i}^{k+1}=\delta \;\text{shrink}\left(v_{i}^{k+1},\mu \right), \end{array}$$

where the shrinkage operator is defined as follows:

(13) $$\begin{array}{c} \text{shrink}\left(x,\gamma \right)=\frac{x}{\left|x\right|}*\max\left(\left|x\right|-\gamma ,0\right). \end{array}$$

The main outline of the linearized Bregman algorithm with sparse regularization is given in Algorithm 1, and, as for the stopping condition, we choose ‖*S* ^*k*+1^ − *S* ^*k*^‖/‖*S* ^*k*^‖ ≤ *ε* = 1.0 × 10^−3^.

## 3. Experimental Results and Discussion

In this section, numerical simulation and* in vivo* experiments were conducted to evaluate the proposed method (LBSR) compared with the *l*_2_-norm regularization method based on the conjugate gradient method (*l*_2_-CG) and *l*_1_-norm regularization method based on the Split Bregman iterative method (*l*_1_-SB) [27] for source reconstruction, respectively. All of the reconstruction results were conducted on a personal computer using MATLAB R2010a, with Intel Core CPU 2.53 GHz and 4.00 GB RAM.

We demonstrate the efficiency of the reconstruction by the proposed method, which was quantitatively performed in terms of location error (LE), and LE could be defined as $LE=\sqrt{{\left(x-x_{0}\right)}^{2}+{\left(y-y_{0}\right)}^{2}+{\left(z-z_{0}\right)}^{2}}$, where (*x*_0_, *y*_0_, *z*_0_) is the actual source center and (*x*, *y*, *z*) is the reconstructed source center. The reconstructed time and source energy were used to evaluate the reconstruction performance. In addition, the contrast-to-noise ratio (CNR) was also used as metrics to evaluate whether the reconstructed results could be clearly distinguished from the background, and larger CNR means better performance [33, 34].

### 3.1. Heterogeneous Phantom Stimulation

**Figure 1:**
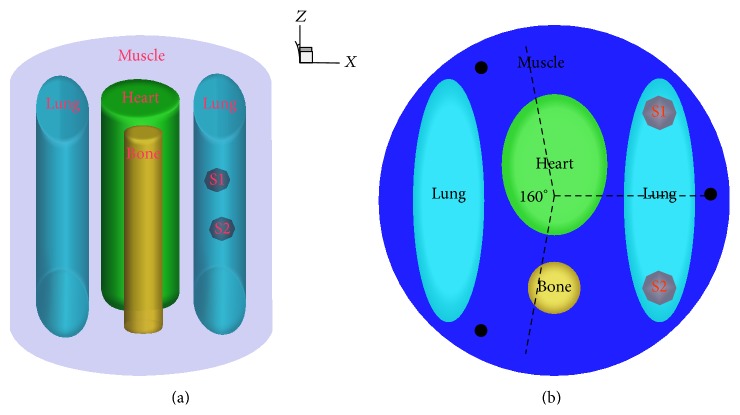
A heterogeneous cylindrical phantom. (a) 3D view of the phantom; (b) cross section of the phantom in the *z* = 0 plane.

Heterogeneous phantom stimulation experiments were conducted to test the performance of the proposed method. The heterogeneous cylindrical phantom was 20 mm in diameter and 20 mm in height, which included the two sources S1 (6.00, 5.00, 0.00) and S2 (6.00, −5.00, 0.00), and also included four types of materials to represent muscle (M), lungs (L), bone (B), and heart (H) as shown in Figure 1. The corresponding optical parameters of different tissues for both the excitation and emission wavelength were set as listed in Table 1 [29]. The phantom was discretized into 5657 nodes and 30676 tetrahedral elements, and the 3D view of the phantom and cross section of the phantom in the *z* = 0 plane are also shown in Figures 1(a) and 1(b), respectively. The black dots in Figure 1(b) represent the excitation light sources, which were modeled as isotropic point sources located in the *z* = 0 plane. To generate the fluorescence measurements, for each excitation source, the emitted fluorescence was captured from the opposite side of the cylindrical model with a 160° field of view as illustrated in Figure 1(b).

**Figure 2:**
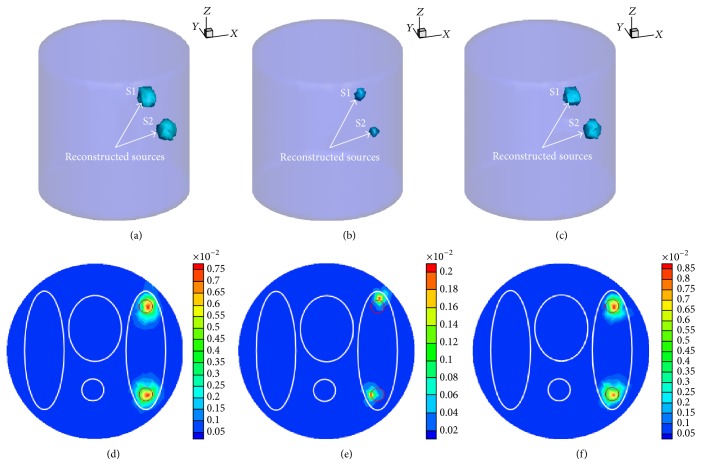
Reconstruction results using different methods. ((a) and (d)) Reconstruction results based on the *l*_2_-CG method; ((b) and (e)) reconstruction results based on the *l*_1_-SB method; ((c) and (f)) reconstruction results based on the LBSR method. Top row: 3D views of the reconstruction results. Bottom row: the corresponding slice image reconstruction results in the *z* = 0 plane. The red circles in the slice images denote the real locations of the sources.

In order to better evaluate the performance of the proposed method, we compared our method with another two classical and effective reconstruction methods to reconstruct the same data while maintaining the same termination condition. *l*_2_-CG is a very classical method based on *l*_2_-norm regularization by the conjugate gradient method to solve the ill-posed inverse problem. *l*_1_-SB is also a very effective method based on sparse regularization because of the introduction of compressed sensing with the Split Bregman iterative method to solve this kind of constrained optimization problem.  Figure 2 shows the reconstruction results by the three methods including 3D views and the corresponding slice image reconstruction results. From the reconstruction results, it emerges that *l*_2_-CG can reconstruct an accurate source location, but it can produce oversmooth results and have more scattering in the two source regions and it also needs more time to run as shown in Figure 2(d) and in Table 2. It can be seen that *l*_1_-SB is faster than *l*_2_-CG method, and the reason is that the main idea of the Split Bregman method is to decompose a complex optimization problem to two independent suboptimization problems by introducing an auxiliary variable in order to make it easy to implement. But the reconstructed sources are sparser and are not accurately localized with a location error of 1.00 mm as shown in Figure 2(e). In contrast, the proposed method is very accurate for source reconstruction and the reconstruction time is very small, which demonstrates that the proposed method is effective. In addition, the proposed method also has good reconstructed source energy, and the reconstructed quantitative results are shown in Table 2.

### 3.2. *In Vivo* Mouse Experiments

**Figure 3:**
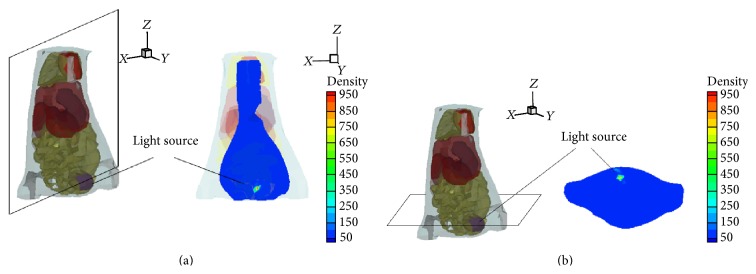
The biodistribution of I-131 uptake in the heterogeneous mouse bladder. (a) Coronal view of the bladder; (b) cross section of the bladder in the *z* = 3.68 plane.

To further validate the feasibility of the proposed method in practical application, an* in vivo* experiment on an athymic nude mouse was performed with a dual-modality optical and micro-CT imaging system previously developed by our group [35–37], which was to acquire the Cerenkov luminescence data and anatomical structural data by a cooled, sensitive charge-coupled device (CCD) and micro-CT, respectively. The* in vivo* mouse data is provided by Hu et al. [38], and the main process of* in vivo* mouse experiments can be summarized as follows. First, the mouse was injected with intravenous tail injection and the injected doses of Iodine-131 (I-131) were 400 *μ*Ci, which were performed for the 3D reconstruction of I-131 uptake in the mouse bladder. Second, two hours later, the raw micro-CT data and Cerenkov luminescence data were acquired by dual-modality imaging system. In order to build the heterogeneous mouse model, the organs of micro-CT data were segmented, which included adipose, bladder, heart, lungs, liver, spleen, stomach, kidneys, bone, and intestines, and we need to integrate them into one volume of data as shown in Figure 3. The corresponding optical parameters of the biological tissues are listed in Table 3 [38]. Finally, the heterogeneous mouse torso was discretized into 3718 nodes and 18952 tetrahedrons for the reconstruction. In addition, the geometrical center of the bladder was defined as the actual source location, which could be obtained by micro-CT images at 18.24, 25.76, and 3.68 mm.

**Figure 4:**
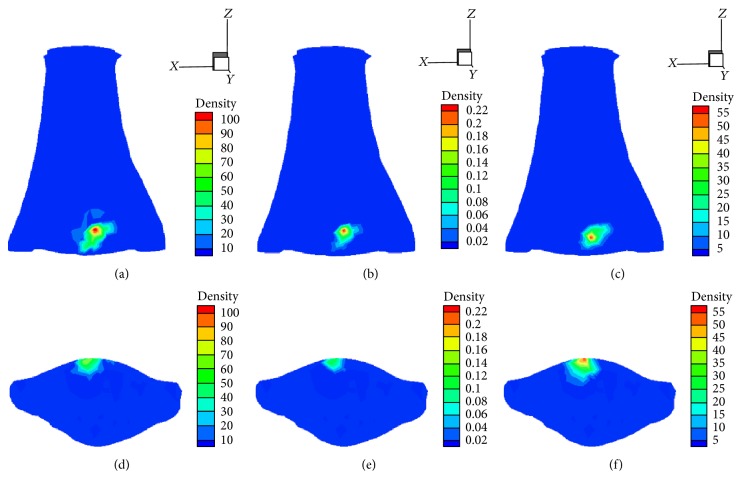
Reconstruction results using different methods. ((a) and (d)) Reconstruction results based on the *l*_2_-CG method; ((b) and (e)) reconstruction results based on the *l*_1_-SB method; ((c) and (f)) reconstruction results based on the LBSR method. Top row: coronal views of the reconstruction results. Bottom row: the corresponding cross section reconstruction results in the *z* = 3.68 plane.

The 3D reconstruction of I-131 uptake in the mouse bladder was performed using *l*_2_-CG method, *l*_1_-SB method, and LBSR method including horizontal and coronal views of the bladder as shown in Figure 4. The three methods produced almost the same results in the first two coordinates of the actual location, but the third coordinate location reconstructed from the proposed method was completely different from the other two methods. In addition, the reconstructed error was 1.82 mm, 1.82 mm, and 0.15 mm by *l*_2_-CG method, *l*_1_-SB method, and LBSR method, respectively, as shown in Table 4, which indicated that the LBSR method was very accurate under the very ill-posed linear equation. In addition, the computation time of all the methods was small, but the proposed method is still faster than the other two methods because the permissible source region *I*_*x*_ × *I*_*y*_ × *I*_*z*_ = [10,25]×[15,35]×[2,10] was exploited, which could reduce the size of the system matrix. Meanwhile, the permissible source region also reduces the ill-posedness and improves the reconstruction quality [13, 14]. The quantitative comparisons of the reconstruction results for the above three methods are given in Table 4. *l*_2_-CG method produced some scattering and artifacts, and *l*_1_-SB method produced sparse source as shown in Figures 4(a) and 4(b), respectively. However, the reconstructed source was more concentrated and had a good contrast with the background by using the LBSR method as shown in Figure 4(c). In addition, it can be noted that the LBSR method is accurate based on the source distribution from Figure 4 and Table 4, which indicates that the LBSR method has an advantage in accuracy for practical* in vivo* applications.

## 4. Conclusion 

It is well known that the quality of reconstructed images largely depends on the reconstruction algorithm. In this paper, we proposed an effective algorithm based on the linearized Bregman method with sparse regularization for reconstruction. The linearized Bregman iteration method is exploited to minimize the sparse regularization problem, which requires little computation time and can accelerate the convergence process so as to further achieve fast and accurate source reconstruction. The numerical simulation and* in vivo* mouse experiment were used to evaluate the performance of the proposed method and the other two methods. The experiment results indicate that *l*_2_-CG method could produce some scattering and a smooth solution, and *l*_1_-SB method produced sparse source and created some big errors. In contrast, the proposed method is accurate and efficient for reconstruction. Future work will focus on studying the reason why the error is relatively big of the second coordinate location reconstructed for* in vivo* experiments and further improve the proposed method for more practical applications such as early detection of tumor and evaluation of treatment.

## Figures and Tables

**Algorithm 1:**
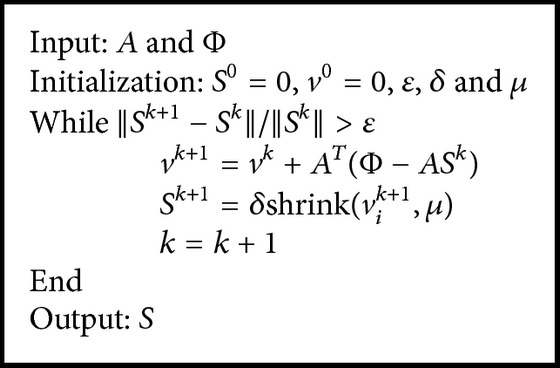
Linearized Bregman algorithm with sparse regularization (LBSR).

**Table 1:** Optical parameters for each organ in the heterogeneous cylindrical phantom.

Coefficient	Muscle	Lungs	Bone	Heart
*μ*_*ax*_ [mm^−1^]	0.0052	0.0133	0.0024	0.0083
*μ*_*sx*_′ [mm^−1^]	1.08	1.97	1.75	1.01
*μ*_*am*_ [mm^−1^]	0.0068	0.0203	0.0035	0.0104
*μ*_*sm*_′ [mm^−1^]	1.03	1.95	1.61	0.99

**Table 2:** Quantitative results for two sources by different methods.

Methods	Reconstructed position center (mm)	LE (mm)	CNR	Reconstruction time (s)	Maximum reconstructed energy value (nW/mm^3^)
*l*_2_-CG	*S*1 (6.00, 5.00, 0.00) *S*2 (6.00, −5.00, 0.00)	0.00 0.00	13.2	223.58	0.0022 0.0019

*l*_1_-SB	*S*1 (6.00, 6.00, 0.00) *S*2 (5.00, −5.00, 0.00)	1.00 1.00	17.6	72.18	0.0080 0.0079

LBSR	*S*1 (6.00, 5.00, 0.00) *S*2 (6.00, −5.00, 0.00)	0.00 0.00	20.8	6.68	0.0091 0.0082

**Table 3:** Optical parameters of biological tissues for the mouse organ regions.

Coefficient	Adipose/bladder	Heart	Lungs	Liver/spleen	Stomach	Kidneys	Bone	Intestines
*μ*_*a*_ [mm^−1^]	0.1017	1.5477	4.6832	9.2860	0.3082	1.7334	1.5233	0.2891
*μ*_*s*_′ [mm^−1^]	1.2929	1.1674	2.3271	0.7786	1.6320	2.7599	3.0393	1.3548

**Table 4:** Quantitative results for one source by different methods.

Methods	Actual source location (mm)	Reconstructed position center (mm)	Reconstruction time (s)
*l*_2_-CG	(18.24, 25.76, 3.68)	(18.82, 29.90, 5.50)	0.6342
*l*_1_-SB	(18.24, 25.76, 3.68)	(18.82, 29.90, 5.50)	0.5063
LBSR	(18.24, 25.76, 3.68)	(19.06, 29.98, 3.83)	0.4853
